# A Rare Case of Breast Metastasis from a Primary Lung Tumor: Case Report

**DOI:** 10.3390/curroncol31080350

**Published:** 2024-08-16

**Authors:** Raquel Diaz, Federica Murelli, Letizia Cuniolo, Chiara Cornacchia, Francesca Depaoli, Cecilia Margarino, Chiara Boccardo, Marco Gipponi, Simonetta Franchelli, Marianna Pesce, Barbara Massa, Silvia Bozzano, Valentina Barbero, Franco De Cian, Piero Fregatti

**Affiliations:** 1 Department of Surgical and Diagnostic Integrated Sciences—DISC, University of Genova, 16132 Genova, Italy; 2 Breast Surgery, IRCCS Ospedale Policlinico San Martino, 16132 Genova, Italy; 3 Anatomic Pathology Unit, IRCCS Ospedale Policlinico San Martino, 16132 Genova, Italy; 4 Department of Internal Medicine and Medical Specialties, IRCCS Ospedale Policlinico San Martino, 16132 Genoa, Italy

**Keywords:** breast cancer, breast metastasis, lung cancer

## Abstract

Breast metastasis originating from a primary lung tumor is exceedingly rare and can present challenges in distinguishing it from primary breast cancer. This case report discusses the management of a 64-year-old woman who initially presented with a nodule in her left breast. A biopsy revealed an infiltrating ductal carcinoma. Despite negative BRCA genetic testing, her significant family history of cancer and the presence of a newly detected right breast lesion led to a bilateral mastectomy. Post-operative imaging identified multiple hypodense nodules and a spiculated pulmonary nodule, necessitating further investigation. An endoscopic lung biopsy confirmed a primary pulmonary carcinoma with histological features similar to the breast carcinoma, suggesting the lung as the primary source. This case highlights the complexity of differentiating breast metastasis originating from a lung tumor and primary breast cancer. It underscores the importance of comprehensive diagnostic evaluations and the consideration of extramammary origins in metastatic cases. The findings emphasize the role of multidisciplinary teams in managing such rare and challenging cases and highlight the necessity for thorough and repeated assessments in atypical breast cancer presentations.

## 1. Introduction

Breast metastases originating from a primary lung tumor are exceedingly rare and present a significant diagnostic challenge [1]. Most breast metastases typically arise from cancers such as melanoma, lymphoma, or gynecological malignancies, making lung carcinoma an uncommon source [2]. These cases necessitate a comprehensive and multidisciplinary diagnostic approach to accurately differentiate primary breast cancer from metastatic disease. Accurate diagnosis is essential for determining the most effective treatment strategy and assessing the prognosis. This case report details the diagnostic process and multidisciplinary management of a 64-year-old woman who presented with a nodular formation in her left breast, which was later confirmed to be a breast metastasis from a primary lung tumor. This case provides rare insight into the metastatic pathways and diagnostic intricacies associated with such occurrences.

## 2. Case Presentation

A 64-year-old woman with no relevant medical history, no drug allergies, and no significant surgical history noticed a nodular formation in the upper outer quadrant of her left breast during self-examination in March 2024. She was initially managed at another institution where she underwent a follow-up mammogram and subsequently a biopsy. Mammography revealed two lesions in the left breast, measuring 7 mm and 8 mm, respectively. Biopsy findings indicated an infiltrating ductal carcinoma with a Ki-67 of 50%, estrogen (ER), progesterone (PR), and Human Epithelial Receptor 2 (HER2)-negative. Therefore, she was referred to the Breast Unit of our Institution.

A multidisciplinary team (MDT) recommended a nipple-sparing mastectomy due to her small breast size, and biopsy of the sentinel lymph node.

During the preoperative evaluation, another mass was detected in the lower inner quadrant of the right breast, which had previously gone unnoticed.

Our breast radiologists re-examined the prior mammographic images and confirmed that the exam of the right side was negative. Thus, they decided to perform a right-sided unilateral breast ultrasound on the patient.

The ultrasound of the right breast revealed a nodular lesion with mixed echogenicity, classified as E4b. The patient had a significant family history of cancer, including leukemia in her father, lung carcinoma in her mother, and breast carcinoma in her paternal aunt. Genetic testing for the BRCA mutation returned negative results.

Despite her family history, she opted for a bilateral mastectomy without further investigation on the right side.

In April 2024, she underwent preoperative tests (blood tests, chest X-ray, and ECG), all of which returned normal results.

Subsequently, she underwent bilateral nipple-sparing mastectomy along with a sentinel lymph node biopsy and immediate breast reconstruction with tissue expanders. Histological examination revealed poorly differentiated neoplasms. Histology of the left breast showed invasive ductal carcinoma pT1c/G3/N0, while the right breast showed invasive ductal carcinoma m(2)pT1b/G3/N0.

Post surgery, she underwent radiological staging examinations to determine the need for adjuvant chemotherapy.

Staging CT scans revealed multiple hypodense nodules with hyperemic rims, mostly on the left side. Additionally, a spiculated-margin pulmonary nodule that was not visible on the preoperative chest X-ray, as well as an adrenal metastasis, was found. Biopsy of the subcutaneous nodules revealed carcinomatous involvement with the expression of cytokeratin 7 and partial CDX2.

Immunohistochemical staining was negative for various markers. The proliferation index was 70–80%. However, the histopathological findings did not conclusively determine the neoplasm’s origin.

Finally, the patient underwent an endoscopic lung biopsy with multiple cryobiopsies of a subsegmental branch of the right upper lobe bronchus, performed under fluoroscopic guidance and radial endobronchial ultrasound (EBUS). 

In May 2024, histological examination confirmed the presence of carcinoma, specifically involving the bronchial mucosa.

**Figure 1:**
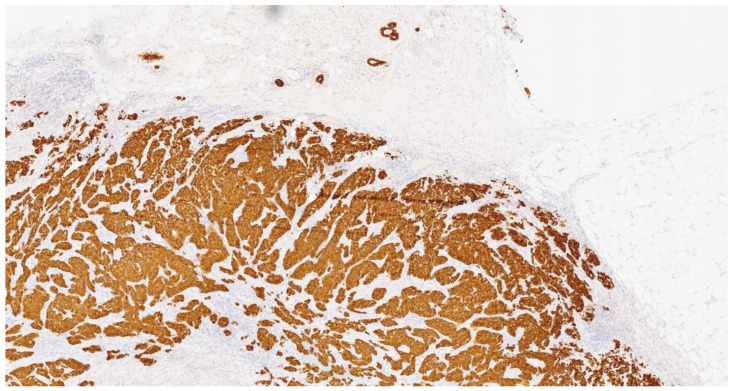
Cytokeratin 7 (CK7). CK7 expression appears diffuse and intense in the neoplasm and in the healthy breast tissue (upper right side).

**Figure 2:**
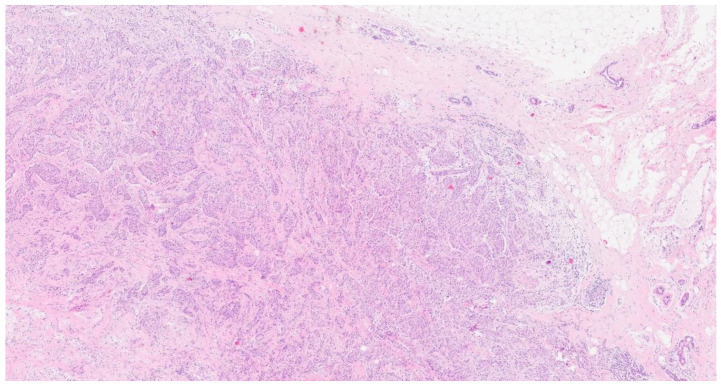
Hematoxylin–eosin 4×. The hematoxylin–eosin stain shows a breast localization (with healthy parenchyma visible in the upper right side) of a poorly differentiated carcinoma with a solid structure composed of epithelial elements with round, vesicular nuclei; small nucleoli; abundant eosinophilic cytoplasm; and a moderate desmoplastic reaction. Numerous mitotic figures are also observed.

The carcinoma exhibited infiltration near the cartilaginous tissue by a neoplasm characterized by solid and glandular growth and the widespread expression of cytokeratin 7 (Figure 1). Overall, the histopathological findings were substantially similar to those observed in the previous histological examination of the breast tissue (Figure 2). Pathologists conducted further examinations on the breast and lung biopsies, testing for TTF1 (Thyroid Transcription Factor-1), the GATA3 gene, napsin A, and P40, a variant of the p63 protein encoded by the TP63 gene. All these examinations returned negative results. PD-L1 (Programmed Death-Ligand 1, a key target in anticancer therapy) was absent, and ALK (Anaplastic Lymphoma Kinase), EGFR (Epidermal Growth Factor Receptor), the ROS1 gene, the RET gene, METex14 (specific mutation in the Mesenchymal–Epithelial Transition factor), NTRK1/2/3 (Neurotrophic Tyrosine Receptor Kinase), and BRAF (B-Raf Proto-Oncogene, Serine/Threonine Kinase) all showed wild-type status (wt) (Table 1).

Considering the radiological findings, histopathological results, and disease progression, the evidence strongly suggests a primary pulmonary origin. Therefore, both the breast subcutaneous neoplasms and adrenal metastasis were deemed to originate from the lungs. Consequently, it was decided to initiate a first-line therapy with carboplatin AUC 5, paclitaxel, and pembrolizumab administered every 3 weeks.

On physical examination, the patient is currently in good general condition, asymptomatic, and with an oxygen saturation of 97% on room air.

**Figure 3:**
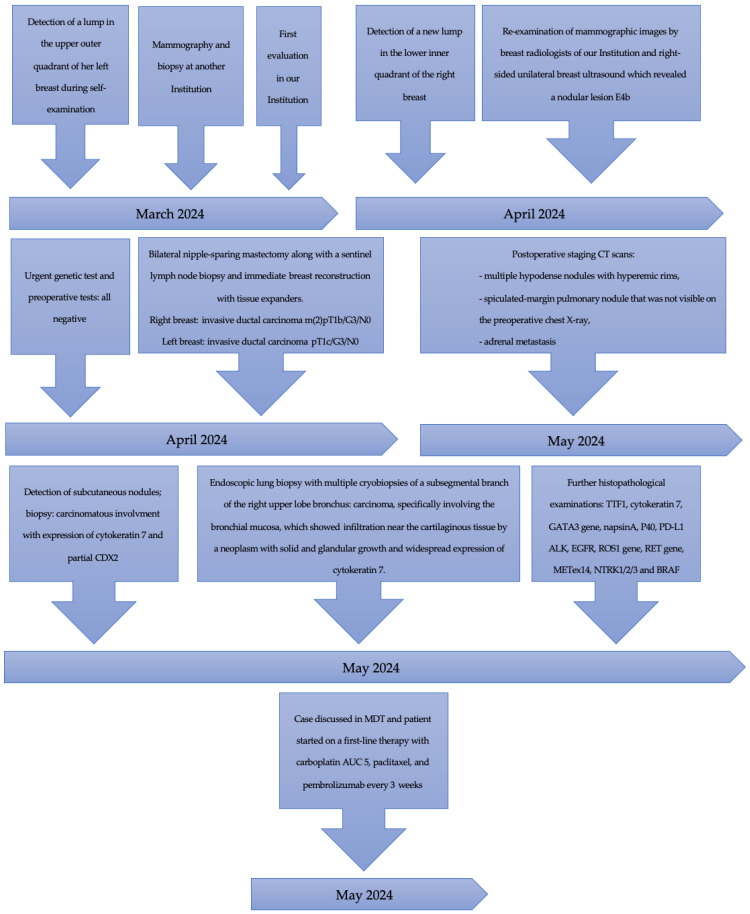
All the important examinations are summarized in this timeline.

All the important examinations are summarized in a timeline (Figure 3).

## 3. Discussion

This case report presents a complex diagnostic and therapeutic challenge involving a 64-year-old woman who presented with a left breast nodule, initially wrongly confirmed as breast cancer with a high proliferation index (Ki-67 of 50%), ER, PR, and HER2-negative, necessitating a multidisciplinary approach. The decision to perform a nipple-sparing mastectomy was influenced by the small breast size.

The discovery of a new lesion in the right breast during preoperative evaluation further complicated the clinical picture. Despite negative genetic testing for BRCA mutations, the patient’s significant family history of various cancers raised concerns about potential genetic predispositions and metastatic disease.

Histological findings from both breasts revealed poorly differentiated neoplasms, prompting further radiological staging. The presence of multiple hypodense nodules with hyperemic rims and a spiculated-margin pulmonary nodule on CT scans raised the suspicion of metastatic disease. The subcutaneous nodules biopsied showed carcinomatous involvement with cytokeratin 7 (CK7 typically expressed in epithelial cancers such as breast, lung, ovarian, and pancreatic cancers), but the immunohistochemical staining was inconclusive regarding the primary origin of the neoplasm.

The definitive diagnosis was established via the endoscopic lung biopsy, which identified a carcinoma involving the bronchial mucosa with infiltration near cartilaginous tissue, displaying both solid and glandular growth patterns. The histopathological findings, which were similar to those seen in the breast tissue, suggested a primary pulmonary origin.

This case highlights several critical points like the potential complexity of breast lesions. The role of family history is crucial in understanding potential genetic predispositions, even when genetic testing is negative. This case underscores the importance of considering metastatic pathways from non-mammary primary sites, especially in patients with unusual presentations or multiple lesions. Furthermore, one of the most important aspects is the multidisciplinary approach [3]. Effective management of such complex cases requires a coordinated effort among oncologists, radiologists, pathologists, and surgeons to ensure accurate diagnosis and optimal treatment planning. 

This was an extremely rare case that, based on the clinical, radiological, and histological data available at the beginning of the referral, might have led to an initial misdiagnosis. However, it emphasizes uncommon clinical presentations, such as the rapid appearance of contralateral neoplasms in addition to those already studied on the left breast. 

In the literature, the most complete article we found was a systematic review by Jennifer A. Mirrielees et al. [1] which reported cases of patients with synchronous or metachronous breast metastases from primary lung tumors. Except for two cases, all identified breast metastases from primary lung tumors had negative estrogen and progesterone receptors, and HER2 was not amplified. The most common treatment history was chemotherapy (CT) for lung cancer alone (36%), 20% received CT + surgery, 16% received surgery alone, 12% received CT + radiotherapy (RT), 4% received RT + surgery, 4% received RT alone, and 8% received none. Finally, more cases of non-small-cell lung cancer (NSCLC) metastases have been documented compared to those of small-cell lung cancer (SCLC) in published case reports. Additional cases have been reported over the last decade, the results of which are summarized in Table 2.

Considering our clinical case and the literature, it is evident that differentiating between primary breast cancer and metastasis from lung cancer can sometimes be challenging.

In summary, the possibility of breast metastasis from lung cancer should be seriously considered in the following scenarios: patients with single or multiple breast lesions without an in situ component; patients with triple-negative breast tumors or with an uncommon histology; breast tumors that present as poorly differentiated and with a particularly aggressive clinical course; cigarette smoking; and a family history of lung cancer [31].

## 4. Conclusions

The diagnosis of a primary pulmonary carcinoma with metastatic involvement of the breasts and subcutaneous tissues is rare but underscores the need for thorough and repeated evaluations in atypical presentations of BC. This case contributes to the understanding of metastatic BC and highlights the necessity of considering extramammary origins in differential diagnoses.

## Figures and Tables

**Table 1:** Pathological examination results.

Molecule	Results
Cytokeratin 7	+
TTF1	−
GATA3	−
Napsin A	−
P40	−
PD-L1	absent
ALK	wt
EGFR	wt
ROS1	wt
RET	wt
METex14	wt
NTRK1/2/3	wt
BRAF	wt

+ = POSITIVE; − = NEGATIVE; wt = WILD-TYPE.

**Table 2:** Review of the literature.

Source	Primary Lung Carcinoma	Gender	Age	Stage	TTF1	ER	PR	HER2
Non-small-cell lung carcinomas								
Babu 2009 [4]	ADCA LCC	Female Female	51 82	IV IV	+ +	− −	− −	− −
Branica 2012 [5]	ADCA	Female	55	NR	+	NR	NR	NR
Chattopadhyay 2012 [6]	SQCC	Female	55	IV	NR	−	−	NR
Choi 2011 [7]	NEC	Female	62	IIA	+	−	−	−
Fukumoto 2011 [8]	ADCA	Female	65	IIIA	+	−	NR	NR
Gomez-Caro 2006 [9]	ADCA	Male	65	IB	−	NR	NR	NR
Hsu 2008 [10]	SQCC	Female	48	IV	−	−	−	NR
Hunter 1993 [11]	LCC	Female	57	III	−	−	−	−
Ji 2012 [12]	ADCA ADCA	Female Female	49 40	IV IV	+ +	− −	− −	− −
Jitendra 2011 [13]	ADCA	Female	42	NR	+	−	−	−
Klingen 2009 [14]	ADCA ADCA	Female Male	79 70	NR NR	+ +	NR NR	NR NR	NR NR
Ko 2012 [15]	ADCA	Female	47	IV	+	−	−	−
Maounis 2010 [16]	ADCA	Female	73	IV	+	−	NR	NR
Noguera 2007 [17]	SC NS	Female Female	41 53	NR NR	NR NR	NR NR	NR NR	NR NR
Rimner 2007 [18]	LCC ADCA	Female Female	49 81	IV NR	NR +	NR −	NR −	NR −
Sadikot 1997 [19]	SPCC	Female	47	IV	NR	NR	NR	NR
Sato 2012 [20]	ADCA	Female	57	IV	+	−	−	−
Sengupta 2012 [21]	SQCC	Female	60	IV	−	−	−	NR
Ucar 2007 [22]	ADCA	Male	63	IV	+	NR	NR	NR
Vaughan 2007 [23]	NEC NEC NEC	Female Female Female	30 35 28	NR IV NR	NR NR NR	NR − NR	NR − NR	NR − NR
Verger 1999 [24]	ADCA	Male	63	IIA	NR	NR	NR	NR
Yeh 2004 [25]	ADCA	Female	44	NR	NR	NR	NR	NR
Yoon 2010 [26]	ADCA	Female	42	IIB	+	−	−	−
Mirrielees 2014 [1]	LCC ADCA	Female Female	67 58	IV IIIA	+ +	NR −	NR −	NR −
Yan-Wei Shen 2015 [27]	ADCA	Female	54	NR	NR	NR	NR	NR
Liyu Cao 2020 [28]	ADCA	Female	55	NR	+	−	−	−
Roshini Ramwani 2022 [29]	ADCA ADCA ADCA	Female Female Female	65 74 65	NR NR NR	NR + +	− NR +	− NR −	− NR −
Gábor Cserni 2017 [30]	ADCA	Female	60	NR	+	+	−	−
Juan Li 2022 [31]	ADCA	Female	44	NR	+	−	+	−
Carmine Valenza 2022 [32]	ADCA	Female	63	IIB	+	+	+	+
Xin Chuang 2018 [33]	ADCA	Female	44	NR	+	−	−	−
Small-cell lung carcinomas								
Altintoprak 2011 [34]	SCC	Male	47	IV	+	−	−	NR
Babu 2009 [4]	SCC	Female	69	IV	+	NR	NR	NR
Courtney 1989 [35]	OCC	Female	59	IV	NR	NR	NR	NR
Jakovijevic 2003 [36]	SCC	Female	44	IV	NR	NR	NR	NR
Kelly 1998 [37]	SCC SCC	Female Female	64 39	IV IV	NR NR	NR NR	NR NR	NR NR
Liu 2009 [38]	SCC	Female	45	IV	NR	NR	NR	NR
Luh 2008 [39]	SCC	Female	50	IV	+	+	+	+
Sharma 2010 [40]	SCC	Female	66	NR	NR	−	−	−
Vaughan 2007 [23]	SCC	Female	83	IV	NR	NR	NR	NR

AC = ANAPLASTIC CARCINOMA; ADCA = ADENOCARCINOMA; ER = ESTROGEN RECEPTOR; HER2 = HUMAN EPITHELIAL RECEPTOR 2; LCC = LARGE-CELL CARCINOMA; NE = WITH NEUROENDOCRINE DIFFERENTIATION; NEC = NEUROENDOCRINE CARCINOMA; NR = NOT REPORTED; NS = NOT SPECIFIED; OCC = OAT CELL CARCINOMA; PR = PROGESTERONE RECEPTOR; SCC = SMALL-CELL CARCINOMA; SQCC = SQUAMOUS CELL CARCINOMA; SPCC = SPINDLE CELL CARCINOMA; + = POSITIVE BY IMMUNOHISTOCHEMISTRY; − = NEGATIVE BY IMMUNOHISTOCHEMISTRY.

## Data Availability

The data presented in this study are available in this article.
